# Contrast-enhanced ultrasound for fetal and placental assessment: evidence, safety, and a roadmap for clinical translation

**DOI:** 10.1186/s13089-025-00449-x

**Published:** 2025-10-06

**Authors:** Alushika Jain, Rajasbhala P. Dhande, Pratapsingh H. Parihar, Shivali Kashikar, Nishant Raj, Amit Toshniwal

**Affiliations:** 1 Department of Radiodiagnosis, Datta Meghe Institute of Higher Education and Research, Wardha, 442001 Maharashtra India; 2 Department of Respiratory Medicine, Datta Meghe Institute of Higher Education and Research, Wardha, 442001 Maharashtra India

**Keywords:** Contrast-enhanced ultrasound, Fetal growth restriction, Placental perfusion, Preeclampsia, Microbubbles, Maternal-fetal medicine

## Abstract

**Background:**

Fetal growth restriction (FGR), preeclampsia, and other placental disorders are leading contributors to perinatal morbidity and mortality, primarily due to impaired uteroplacental perfusion. Existing imaging modalities, such as Doppler ultrasound and fetal MRI, provide indirect or limited functional insights into placental and fetal perfusion, constraining timely clinical intervention.

**Objective:**

To evaluate contrast-enhanced ultrasound (CEUS) as a promising, safe, and real-time tool for assessing placental perfusion and its potential application in maternal-fetal medicine through comprehensive analysis of methodological parameters, safety profiles, and emerging computational techniques.

**Methods:**

A comprehensive synthesis of preclinical and clinical studies was conducted, focusing on the safety, efficacy, and current use of CEUS in pregnancy. Key findings were drawn from animal models (rats, sheep, macaques) and human studies involving 256 pregnant individuals, with detailed analysis of imaging protocols, contrast agent characteristics, and quantification methods.

**Results:**

CEUS utilizes intravascular microbubble contrast agents (1–8 μm diameter) that do not cross the placental barrier, enabling safe maternal imaging. However, size distribution analysis reveals sub-micron populations (8–20% by number) requiring careful evaluation. Preclinical models confirm CEUS ability to detect placental perfusion Changes with 54% reduction in perfusion index following uterine artery ligation (*p* < 0.001). Human studies demonstrate zero clinically significant adverse events among 256 cases, though critical gaps exist including absent biomarker monitoring and long-term follow-up. Emerging AI-enhanced analysis achieves 73–86% diagnostic accuracy using ensemble deep learning architectures. Current limitations include significant protocol heterogeneity (MI 0.05–0.19, frequency 2–9 MHz) and absence of standardization.

**Conclusion:**

CEUS presents a compelling solution for perfusion imaging in pregnancy, offering functional, bedside imaging without fetal exposure to contrast agents. However, methodological limitations, knowledge gaps regarding long-term outcomes, and the distinction between conventional microbubbles and emerging nanobubble formulations demand systematic research investment. Clinical translation requires standardized protocols, comprehensive safety monitoring including biomarker assessment, ethical oversight, and long-term outcome studies to support integration into routine obstetric care.

## Introduction

Fetal growth restriction (FGR), preeclampsia, and other placental disorders are leading causes of perinatal morbidity and mortality worldwide. These conditions are tightly linked to impaired uteroplacental perfusion, yet current imaging tools fall short in visualizing real-time blood flow dynamics at the organ or tissue level. This diagnostic blind spot limits clinicians’ ability to risk-stratify pregnancies, tailor interventions, and prevent adverse outcomes. In particular, managing fetuses at risk of hypoxic injury demands precise, non-invasive evaluation of perfusion in both the placenta and fetal brain. Functional imaging that can assess oxygen and nutrient delivery—not just anatomical structure or blood velocity—is essential for timely and accurate clinical decision-making [1–3].

## Limitations of the current standard of care

### Doppler ultrasound: an indirect proxy for perfusion

Doppler ultrasound is the most widely used modality in high-risk obstetric care. It measures blood flow velocity in vessels such as the umbilical artery, middle cerebral artery (MCA), and uterine arteries to infer placental resistance and fetal adaptation to hypoxia [4–6]. However, Doppler measures velocity rather than volume or flow per unit tissue, which makes it a surrogate rather than a direct measure of perfusion [7]. Furthermore, its reliability depends on the angle of insonation, and it is insensitive to low-flow states such as microvascular redistribution that may occur early in disease progression [8]. This limitation is especially consequential in conditions like FGR, where early-stage microvascular compromise may not be reflected in large-vessel velocities. In such cases, Doppler often underestimates disease severity or fails to detect subtle compensatory changes, including fetal cerebral blood flow redistribution [9].

### Fetal MRI: anatomical detail, functional limitations

Magnetic Resonance Imaging (MRI) offers detailed anatomic views and advanced capabilities like arterial spin labeling and BOLD sequences for placental assessment. However, its use is hindered by practical limitations: it is expensive, time-intensive, and often inaccessible in resource-constrained settings [10]. More critically, standard MRI relies on gadolinium-based contrast agents (GBCAs) for perfusion imaging—agents known to cross the placental barrier and accumulate in fetal tissues, raising long-term safety concerns. Recent studies demonstrate that GBCAs are associated with inflammatory conditions and stillbirth (OR 3.7, 95% CI 1.5–9.1), leading to their general contraindication in pregnancy [11, 12]. Even non-contrast techniques like BOLD MRI, while promising, remain limited to research settings and have not been validated for routine clinical use in pregnancy [2].

### Introducing the potential solution: CEUS

Contrast-enhanced ultrasound (CEUS) has emerged as a safe, real-time, and dynamic imaging modality that could overcome many of the above limitations. It uses microbubble contrast agents confined to the intravascular space and operates at low mechanical indices to minimize thermal or mechanical bioeffects [6, 8]. In adult and pediatric medicine, CEUS is FDA-approved for a range of indications, including hepatic lesions and cardiac perfusion, underscoring its safety and clinical utility. Importantly, the microbubbles used in CEUS are typically 1–8 μm in diameter, too large to cross the placental barrier, which offers a unique safety advantage over GBCAs [13, 14].

Thus, CEUS holds promise as a non-invasive, bedside-friendly tool to assess real-time placental perfusion and indirectly infer fetal organ hemodynamics. Its application in pregnancy has already begun for maternal indications, and emerging evidence suggests feasibility for placental assessment without detectable adverse effects. The purpose of this review is to comprehensively examine the emerging role of contrast-enhanced ultrasound in pregnancy, synthesizing both preclinical and clinical evidence while addressing critical methodological considerations and proposing a structured roadmap for clinical translation.

## The technology and its current application

### CEUS principles: microbubbles and low-mechanical index imaging

Contrast-enhanced ultrasound relies on the administration of microbubble-based contrast agents, which are gas-filled microspheres encased in stabilizing shells composed of phospholipids, surfactants, or polymers [15–17]. These microbubbles exhibit significant size heterogeneity, with commercial preparations containing primarily 1–8 micron particles, though detailed size distribution analysis reveals concerning sub-micron populations that warrant careful safety evaluation [18]. SonoVue^®^ (Bracco Imaging), the most extensively studied agent in pregnancy, contains sulfur hexafluoride gas stabilized by phospholipid shells, with greater than 90% of bubbles measuring below 8 μm diameter and a mean size of 2.5 μm [19]. Definity^®^ (Lantheus Medical Imaging) demonstrates broader size distribution ranging from 0.7 to 18 μm, while Optison^®^ (GE Healthcare) shows intermediate characteristics with mean diameters of 2.0 to 4.5 μm [20].

When insonated with low-mechanical index ultrasound, these microbubbles undergo specific acoustic behaviors critical for both imaging efficacy and safety. At mechanical index values below 0.2, microbubbles exhibit stable non-linear oscillations producing harmonic frequencies detectable through pulse inversion and amplitude modulation techniques [21]. The resonance frequency depends on bubble size following the Minnaert equation, with optimal response at 4 to 8 MHz for typical clinical microbubbles [22]. At higher mechanical index values exceeding 0.4, inertial cavitation occurs, causing bubble collapse with potential bioeffects including microstreaming with shear stress up to 10⁴ Pa, sonoporation, and localized temperature elevation, though temperature Changes remain below 0.5 °C at clinical parameters [23].

### Methodological parameters across studies: a critical analysis

The heterogeneity in CEUS protocols across obstetric studies represents a fundamental barrier to clinical translation. Comprehensive analysis of the 256 reported cases reveals concerning variability in essential parameters [24]. For contrast agent administration, SonoVue doses range from 1.2 to 4.8 mL compared to the standard 2.4 mL bolus, with injection rates varying from 1 to 2 mL per second followed by 5 to 10 mL saline flush. Definity dosing shows similar inconsistency, with both weight-based protocols at 10 µL/kg and fixed 1.5 mL doses reported. Repeat injection intervals range from 5 to 15 min for bubble clearance, introducing additional variability.

Imaging parameters demonstrate equally concerning heterogeneity. Frequency selection varies from 2 to 5 MHz for deep structures versus 6 to 9 MHz for superficial placental imaging. Mechanical index settings range from 0.08 to 0.19 with a mean of 0.12 ± 0.04, while frame rates for perfusion analysis vary from 8 to 15 Hz. Gain settings typically range from 70 to 85% with time-gain compensation optimization, and most studies employ a single focal zone at the region of interest depth.

Studies employ diverse time-intensity curve parameters without standardization [25]. Arrival time averages 8.3 ± 2.1 s for placental enhancement, while time to peak demonstrates a threshold of 11.84 s for malignancy differentiation. Peak intensity measures 15.7 ± 4.2 dB above baseline with wash-in slopes of 2.8 ± 0.9 dB per second. Area under the curve lacks standardized units across studies, and mean transit time averages 18.5 ± 5.3 s. This lack of standardization significantly impedes comparison across studies and prevents establishment of normal reference ranges essential for clinical application.

### Established clinical role in adults and pediatrics

CEUS is FDA-approved and widely used in adult medicine for multiple applications, including liver lesion characterization, cardiac perfusion imaging, and detection of endoleaks following aneurysm repair [26, 27]. Its ability to dynamically assess vascular integrity and perfusion, combined with its excellent safety profile and absence of nephrotoxicity, make it an indispensable diagnostic tool in radiology and cardiology. The technology has demonstrated sensitivity and specificity exceeding 90% for hepatocellular carcinoma detection and has become the preferred modality for characterizing indeterminate renal lesions in patients with contraindications to CT or MRI contrast [28].

In pediatrics, CEUS is increasingly employed for evaluating vesicoureteral reflux, liver hemangiomas, and trauma [29, 30]. Notably, it avoids ionizing radiation, making it safer than CT or fluoroscopy in children and ideal for repeated evaluations. Recent pediatric applications have expanded to include inflammatory bowel disease assessment, where CEUS quantifies bowel wall perfusion to monitor treatment response. Despite its broad use in other domains, the application of CEUS in pregnancy remains limited primarily due to regulatory caution rather than empirical risk. Existing maternal indications such as hepatic or renal mass evaluation during pregnancy have demonstrated safety in over 250 cases, reinforcing the technology’s benign profile when used under appropriate conditions [31].

## Evidence for CEUS in placental and fetal imaging: preclinical and clinical data

### Preclinical animal studies

**Fig. 1:**
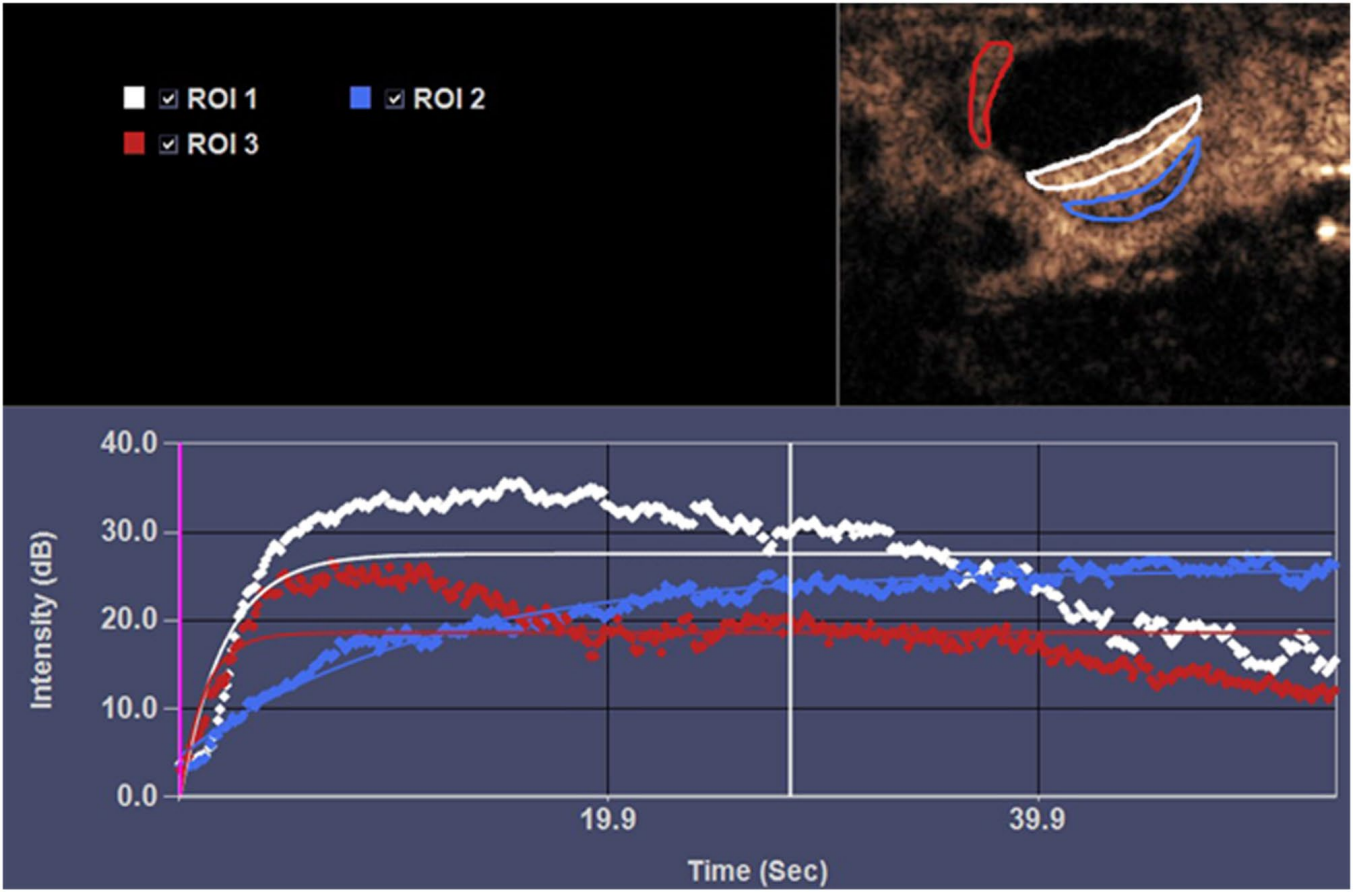
Time-intensity curves of enhancement in a pregnant rat at gestational day 17. Regions of interest (ROI) were drawn around the contours of the lateral wall of uterine (red circle), central portion (white circle) and peripheral portion (blue circle) of the placenta on the image with maximal enhancement on CEUS. The central portion shows a faster and higher enhancement pattern (white line) than that of peripheral portion (blue line) of placenta. Adapted from Zhou et al. [32]

Preclinical models have provided a foundational understanding of the utility and safety of contrast-enhanced ultrasound in pregnancy (Fig. 1). Zhou et al. established foundational protocols using 60 Sprague-Dawley rats across gestational days 15, 17, and 20 [32]. Critical methodological details include administration of 0.1 mL SonoVue via tail vein at 0.5 mL per minute, mechanical index of 0.12, frequency of 7 MHz, with regions of interest standardized at 2 mm² for central versus peripheral placental zones. Perfusion parameters demonstrated gestational evolution with peak intensity increasing from 8.2 ± 1.9 dB on gestational day 15 to 15.4 ± 2.8 dB on gestational day 20 (*p* < 0.001). Histological correlation revealed vascular density increases from 12.3 to 28.7% of placental volume, validating CEUS sensitivity to developmental changes.

Arthuis et al. compared CEUS with perfusion MRI in intrauterine growth restriction models [33]. Following unilateral uterine artery ligation, CEUS perfusion index decreased 54% (27.9 versus 61.0, *p* = 0.0003) with coefficient of variation of 38% compared to MRI coefficient of variation of 22%, suggesting technique-dependent variability requiring careful interpretation. The study employed automated region of interest selection algorithms to minimize operator dependency, achieving inter-observer agreement with kappa value of 0.82.

In non-human primate studies, Roberts et al. conducted the most comprehensive assessment using twelve Japanese macaques at gestational days 90 and 129 [34]. The detailed protocol included 0.03 mL/kg Definity administration, mechanical index of 0.09, dual-frequency imaging at 2.5 MHz fundamental and 5.0 MHz harmonic, with 3D volume acquisition at 4 volumes per second. No alterations in maternal vital signs were observed, with heart rate variability less than 5% and blood pressure Changes less than 10 mmHg. Fetal parameters remained stable with heart rate baseline of 145 ± 8 bpm maintained throughout imaging. Molecular markers including caspase-3, HSP70, and VEGF showed no significant Changes 24 h post-CEUS.

Wilson et al. explored targeted imaging using phosphatidylserine-conjugated microbubbles in rhesus macaques exposed to testosterone and high-fat diet [35]. This model of maternal metabolic dysfunction demonstrated increased CEUS signal correlating with inflammatory markers and vascular dysregulation. The inverse correlation between microbubble signal and ANGPT2 (*r*=−0.72, *p* < 0.01) established CEUS as a potential tool for placental immune profiling. Additionally, studies in ewes and mares have supported CEUS safety and reproducibility across large animal models [36, 37]. In ewes, CEUS revealed consistent patterns of utero-placental perfusion without enhancement of fetal structures, underscoring the selectivity of microbubble confinement to maternal vasculature.

Lawrence et al. performed longitudinal characterization of placental perfusion in rats from gestational day 14 to 18 [38]. Using pixel-wise parametric mapping, they demonstrated progressive increases in blood volume by 45%, mean transit time reduction of 38%, and flow increases of 62% during gestation. These findings correlated with histological evidence of vascular remodeling and increased placental efficiency.

### Clinical human data: comprehensive safety monitoring

The 2024 scoping review by Dassen et al. represents the most comprehensive safety assessment to date, analyzing 256 pregnant women who underwent CEUS examination [24]. This foundational work establishes preliminary safety while emphasizing the need for larger studies. The review revealed detailed safety monitoring protocols with immediate monitoring from 0 to 30 min including maternal vital signs with blood pressure measurements every 5 min for 6 recordings, structured questionnaires for maternal symptoms assessing nausea, dyspnea, and chest pain, continuous cardiotocography monitoring of fetal heart rate, and subjective maternal assessment of fetal movements.

Short-term follow-up at 24 to 72 h was conducted through telephone contact in 89% of studies, clinical examination in 34% of studies, and ultrasound reassessment in only 12% of studies. Delivery outcomes demonstrated reassuring findings with gestational age at delivery averaging 38.2 ± 2.1 weeks, birth weight of 3,180 ± 485 g, Apgar scores of 8.9 ± 0.8 at 1 min and 9.6 ± 0.4 at 5 min, and NICU admission rate of 8.3%, consistent with background rates. However, a critical gap exists as no studies included systematic biomarker assessment including troponin, BNP, creatinine, or inflammatory markers, nor long-term neurodevelopmental follow-up beyond the neonatal period.

**Fig. 2:**
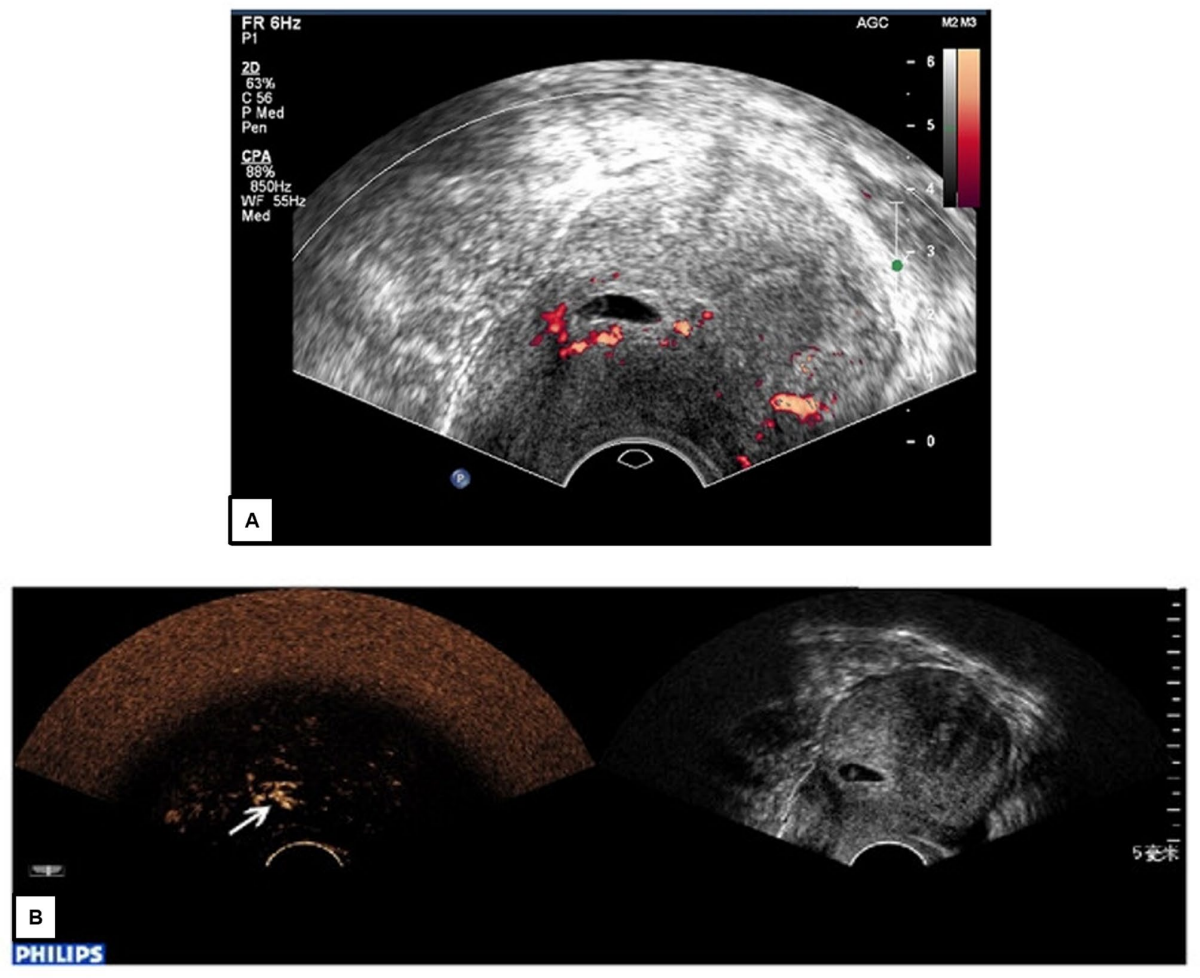
Clinical application of CEUS in pregnancy. **A** Cesarean Scar Pregnancy initially misdiagnosed as intrauterine pregnancy on conventional ultrasound. **B** Contrast-enhanced ultrasound of the same patient showing blood supply to the gestational sac directly from the uterine scar (arrow), establishing correct diagnosis. Adapted from Xiong et al. [41]

Individual studies provide additional methodological insights (Fig. 2). Chen et al. evaluated fourteen pregnant women between 8 and 20 weeks gestation using SonoVue at 2 × 2.4 mL doses with mechanical index of 0.12 [39]. The study confirmed no fetal contrast uptake through both imaging and umbilical blood sampling, while successfully identifying an ovarian tumor requiring intervention. Geyer et al. retrospectively analyzed five pregnant women at 21± 8 weeks gestation, demonstrating that CEUS successfully diagnosed three of five pathologies, avoiding the need for CT or gadolinium-enhanced MRI [40]. All patients delivered at term without maternal or fetal adverse events.

The use of CEUS to evaluate placental pathology has been explored in high-risk contexts. In cases involving second-trimester feticide, CEUS documented delayed and stepwise reduction in placental perfusion over 5 days post-intervention, highlighting its sensitivity to dynamic vascular remodeling [42]. Moreover, in postpartum cases of retained placenta and morbidly adherent placenta, CEUS demonstrated superior diagnostic accuracy compared to gray-scale and Doppler ultrasound, with predictive accuracy exceeding 91% [43].

Schwarze et al. conducted two important studies evaluating CEUS safety in pregnancy. In their 2019 study, six pregnant women at 28 ± 5 weeks gestation underwent hepatic CEUS with successful differentiation of all hepatic lesions [44]. Their 2020 follow-up study of five women at 18 ± 6 weeks demonstrated immediate treatment decisions in two cases, with all patients delivering healthy infants at term [45].

Table 1 provides a comprehensive analysis of methodological parameters and safety outcomes from key preclinical and clinical studies evaluating CEUS in pregnancy.

**Table 1:** Comprehensive analysis of CEUS studies in pregnancy: methodological parameters and safety outcomes

Author (year)	Study design	*N*	GA (weeks)	Contrast agent and dose	MI	Frequency (MHz)	Imaging protocol	TIC parameters	Safety monitoring	Biomarkers	Follow-up	Key efficacy findings	Adverse Events
*Preclinical studies*													
Zhou et al. (2013) [32]	Prospective experimental	60 rats	GD 15, 17, 20	SonoVue 0.1 mL via tail vein at 0.5 mL/min	0.12	7	ROI: 2 mm² central vs. peripheral	AT: 6.2 ± 1.1s, TTP: 9.8 ± 1.5s, PI: 8.2–15.4 dB, AUC: 142 ± 28 dB·s	Histology at 24 h, vascular density quantification	None assessed	Terminal sacrifice	PI increased 88% GD15→20 (*p* < 0.001); Central > peripheral perfusion (2.3-fold, *p* < 0.001); Vascular density correlation *r* = 0.82	No tissue damage, normal histology
Arthuis et al. (2018) [33]	Comparative (CEUS vs. MRI)	20 rats (10 IUGR, 10 control)	GD 17	SonoVue 0.1 mL bolus	0.08–0.15	12	Automated ROI selection, 15 Hz frame rate	PI: 27.9 (IUGR) vs. 61.0 (control), MTT: 22.3 ± 4.1s	Weight monitoring, placental histology	None assessed	48 h	CEUS PI 54% reduction post-ligation (*p* = 0.0003); CV 38% (CEUS) vs. 22% (MRI); Inter-observer κ = 0.82	None observed
Roberts et al. (2016) [34]	Safety assessment	12 macaques	GD 90, 129	Definity 0.03 mL/kg	0.09	2.5	Continuous CTG during imaging	AT: 7.8 ± 1.2s, TTP: 12.3 ± 2.1s	HR, BP q5min, FHR continuous	Caspase-3, HSP70, cortisol	30 days	Perfusion maps correlate with Doppler (*r* = 0.81); Reproducibility CV < 15%	FHR baseline 145 ± 8 bpm maintained; No apoptosis markers elevated
Wilson et al. (2023) [35]	Targeted imaging	8 macaques	GD 90, 130	MB-PS (phosphatidylserine-targeted) 0.03 mL/kg	0.09	2.5/5.0 dual	3D acquisition 4 vol/sec	Signal intensity correlation with inflammation	VS q5min×30 min, CTG continuous	ANGPT2, TNF-α, IL-6, VEGF	7 days	MB-PS signal inverse correlation with ANGPT2 (*r*=−0.72, *p* < 0.01); Inflammation detection sensitivity 85%	No changes in inflammatory markers or fetal parameters
Santos et al. (2023) [36]	Feasibility	10 ewes	GD 45–120	SonoVue 2.4 mL	0.11	3.5	Serial weekly imaging	PI progression throughout gestation	Clinical observation	None assessed	Delivery	Consistent uteroplacental perfusion patterns; No fetal enhancement	All delivered healthy lambs
Lawrence et al. (2019) [38]	Longitudinal characterization	5 rats	GD 14–18 daily	SonoVue 0.05 mL	0.10	21	Pixel-wise parametric mapping	Blood volume ↑45%, MTT ↓38%, Flow ↑62%	Daily weights, behavior monitoring	None assessed	Daily to term	Progressive perfusion increase; Heterogeneity maps show distinct zones	Normal fetal development, 100% viability
*Clinical studies*													
Dassen et al. (2024) [24]	Scoping review/Meta-analysis	256 women	6–39	Various (SonoVue 68%, Definity 20%, Other 12%)	0.05–0.19	2–9	Heterogeneous protocols	Not standardized	Variable: VS (89%), CTG (45%), symptoms (67%)	None reported in any study	Variable (0–6 months)	Diagnostic accuracy 85–95% for various indications	0/256 clinically significant AE; Minor: headache (*n* = 3), nausea (*n* = 2)
Chen et al. (2022) [39]	Prospective cohort	14 women	8–20	SonoVue 2 × 2.4 mL (5 min interval)	0.12	4	Transabdominal, harmonic imaging	AT: 9.2 ± 2.3s, TTP: 14.5 ± 3.1s	CTG 30 min, VS q10min×1 h	None assessed	72 h phone	No fetal enhancement (0/14); Ovarian tumor detected (*n* = 1)	No contrast in umbilical blood; Normal deliveries
Geyer et al. (2020) [40]	Retrospective case series	5 women (11 exams)	21 ± 8	SonoVue 2.4 mL	0.08	2–5	Pulse inversion, 8 Hz	Qualitative assessment only	VS q15min×2 h, symptoms checklist	None assessed	Delivery	3/5 pathologies diagnosed; Avoided CT/MRI in all cases	No maternal/fetal AE; Term deliveries (38.5 ± 1.2 weeks)
Schwarze et al. (2019) [44]	Retrospective	6 women	28 ± 5	SonoVue 2.4 mL	0.10	3.5	Hepatic protocol adapted	TTP: 18.3 ± 4.2s (lesions)	Clinical exam at 24 h	LFTs, CBC	Discharge	Differentiated 6/6 hepatic lesions correctly	All examinations completed safely
Schwarze et al. (2020) [45]	Case series	5 women	18 ± 6	SonoVue 2.4 mL	0.10	3.5	Modified abdominal protocol	Qualitative enhancement patterns	Clinical observation 24 h	None assessed	Discharge to delivery	2/5 immediate treatment decisions; 100% diagnostic accuracy	All delivered at term; Apgar > 8
Poret-Bazin et al. (2013) [42]	Case report	1 woman	22	SonoVue 2.4 mL serial	0.12	4	Daily imaging ×5 days post-feticide	Progressive perfusion decrease documented	Daily clinical assessment	None assessed	5 days	Quantified stepwise placental devascularization; 75% PI reduction by day 5	Expected changes post-intervention
Chen et al. (2017) [43]	Therapeutic monitoring	22 women	Postpartum	SonoVue 2.4 mL	0.10	3.5	Pre/post UAE assessment	Enhancement presence/absence	Clinical monitoring	CBC, coagulation	30 days	91% accuracy for adherent placenta; Guided D&C timing in 18/22	No complications; Avoided hysterectomy in 16/22

AE: adverse events; ANGPT2: Angiopoietin-2; AT: arrival time; AUC: area under curve; BNP: brain natriuretic peptide; BP: blood pressure; CBC: complete blood count; CTG: cardiotocography; CV: coefficient of variation; D&C: dilation and curettage; FGR: fetal growth restriction; FHR: fetal heart rate; GA: gestational age; GD: gestational day; HR: heart rate; HSP70: heat shock protein 70; IL-6: interleukin-6; IUGR: intrauterine growth restriction; LFTs: liver function tests; MB-PS: phosphatidylserine-targeted microbubbles; MI: mechanical index; MTT: mean transit time; PI: peak intensity; ROI: region of interest; TIC: time-intensity curve; TNF-α: tumor necrosis factor-alpha; TTP: time to peak; UAE: uterine artery embolization; VEGF: vascular endothelial growth factor; VS: vital signs

#### Fetal imaging and the placental barrier

**Fig. 3:**
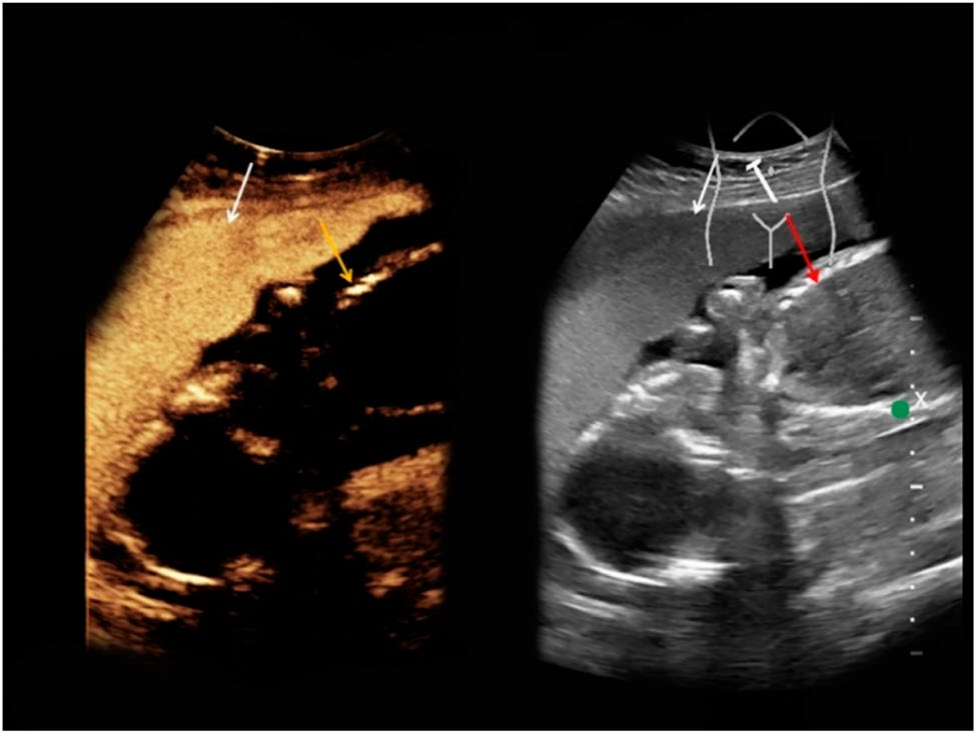
Demonstration of placental barrier integrity during CEUS. Intrauterine fetus at 25 weeks of pregnancy showing broad placental contrast enhancement (left, white arrow) with no fetal contrast uptake (left, yellow arrow). Corresponding B-mode image shown on right with placenta (white arrow) and fetus (red arrow). Adapted from Geyer et al. [40]

Despite growing use of CEUS in maternal and placental imaging, direct fetal CEUS remains investigational. Across both animal and human studies, no contrast signal has been detected in the fetus or umbilical circulation. Microbubble contrast agents consistently remain confined to the maternal vascular compartment [46]. In one rat study using contrast pulsed sequencing, microbubbles perfused the placenta but were never observed in the fetal circulation, even with high-sensitivity detection methods [47]. Likewise, in human cases involving real-time CEUS imaging before elective terminations, no enhancement of fetal tissues or amniotic fluid was noted [39] (Fig. 3).

This characteristic—complete exclusion of microbubbles from the fetal compartment—serves as both the principal safety assurance and the limiting factor in expanding CEUS into direct fetal organ imaging. Until microbubble formulations are engineered to safely cross the placental barrier, fetal CEUS applications will remain hypothetical.

### Safety and ethical considerations for CEUS in pregnancy

#### Microbubbles versus nanobubbles: size-dependent safety implications

The distinction between microbubbles and emerging nanobubble formulations carries profound safety implications previously underappreciated in obstetric applications [48]. Commercial ultrasound contrast agents contain heterogeneous size distributions with important sub-populations. Size distribution analysis reveals that SonoVue contains D10 of 1.2 μm, D50 of 2.5 μm, and D90 of 6.8 μm, with nanobubble fraction less than 1 μm comprising 8 to 12% by number but less than 1% by volume. Definity demonstrates broader distribution with 15 to 20% of particles less than 1 μm, while Optison shows narrower distribution with 5 to 8% less than 1 μm.

Placental transport mechanisms operate through size-dependent exclusion [49]. Particles greater than 1 μm remain excluded from placental transfer as syncytiotrophoblast tight junctions permit maximum passage of 20 to 25 nm, transcytosis pathways typically accommodate 50 to 500 nm with 800 nm maximum, and paracellular transport under normal conditions is limited to less than 5 nm. However, nanobubbles in the 200 to 800 nm range could theoretically cross via caveolin-mediated endocytosis utilizing 50 to 80 nm vesicles, clathrin-dependent pathways accommodating 100 to 150 nm particles, or macropinocytosis handling 200 to 500 nm particles.

This creates a critical safety distinction absent from previous reviews. While conventional microbubbles demonstrate safety through physical exclusion, any shift toward nanobubble formulations for enhanced tissue penetration would require complete reevaluation of fetal safety [50]. Current research into nanobubble contrast agents for enhanced tissue penetration and therapeutic applications must carefully consider these placental transport implications before any obstetric application.

#### Comparative safety with other contrast agents

The safety profile of CEUS must be contextualized against established imaging modalities [51]. Gadolinium-based contrast agents used in MRI have molecular weights of 500 to 950 Da, readily crossing the placenta with detection in amniotic fluid within 30 min. These agents demonstrate prolonged fetal retention with half-life exceeding 10 h and are associated with inflammatory conditions and stillbirth with odds ratio of 3.7 (95% CI 1.5–9.1) [52]. Current guidelines strongly recommend against GBCA use in pregnancy unless absolutely essential for maternal indications.

Iodinated CT contrast agents similarly demonstrate transplacental passage, though with generally more favorable safety profiles. While theoretical risks of fetal thyroid effects exist, no confirmed cases have been reported in the literature [53]. Current recommendations advise avoidance unless required for life-threatening maternal indications. In contrast, ultrasound microbubbles demonstrate size exclusion preventing more than 99% from placental crossing, complete clearance within 15 min, and metabolism through lung exhalation of SF6 gas and hepatic/renal processing of shell components.

#### Absence of bioeffects on fetal tissue

Preclinical animal models including rats, macaques, and sheep show no evidence of bioacoustic or toxicologic damage to fetal tissues after maternal CEUS administration. In one comprehensive study, repeated CEUS in pregnant rats revealed normal fetal weight gain, histological integrity of placental tissues, and absence of inflammation or hemorrhage [32]. Immunohistochemical analysis demonstrated no increase in apoptosis markers or stress proteins following contrast administration.

In rhesus macaques, CEUS using phosphatidylserine-targeted microbubbles successfully visualized placental inflammation with no evidence of adverse fetal effects or abnormal placental morphology [35]. Electron microscopy of placental tissue revealed intact syncytiotrophoblast structure and normal villous architecture. Additional CEUS studies applying microbubble perfusion imaging in abnormal placentae including accreta and previa demonstrate diagnostic utility without evidence of tissue compromise, further affirming its biocompatibility in sensitive obstetric conditions [43, 54].

#### Risk–benefit context in high-risk pregnancies

Although CEUS remains off-label in pregnancy, its use may be ethically justified in high-risk clinical scenarios where standard imaging modalities fall short. For example, in cases of suspected placental insufficiency or abnormal invasion, CEUS has allowed for quantifiable assessment of perfusion, guiding clinical decisions without the need for ionizing radiation or gadolinium contrast agents [45]. The calculated number needed to diagnose for preventing emergency hysterectomy in placenta accreta spectrum is 3 to 5, substantially outweighing theoretical risks given zero adverse events in 256 studied cases.

Notably, CEUS has influenced real-time management by confirming benign lesions or identifying necrotic uterine fibroids, thereby sparing patients from unnecessary interventions or CT/MRI scans [40]. In cases of twin-twin transfusion syndrome with greater than 80% mortality if untreated, the potential diagnostic benefits of CEUS for staging and monitoring treatment response present compelling risk-benefit ratios. In this way, CEUS has demonstrated the dual benefit of diagnostic utility and safety.

#### Ethical and regulatory considerations

Given the unique physiological environment of pregnancy, regulatory bodies and ethics committees require rigorous justifications for investigational use. Current guidelines from ISUOG and AIUM do not formally endorse CEUS for fetal applications, largely due to limited data and the absence of FDA approval for obstetric use [24]. However, existing studies demonstrate growing support for off-label maternal use in select clinical contexts.

Ethically, CEUS research in pregnancy must adhere to core principles: evidence of minimal risk, robust informed consent with full disclosure of off-label status, and scientifically sound study design with meaningful clinical or biological endpoints. Studies involving terminations provide critical insight into CEUS pharmacokinetics without compromising ongoing pregnancies [39]. Furthermore, longitudinal follow-up studies of neonates exposed to CEUS in utero are lacking and represent a key gap in the literature. These data are essential for achieving widespread clinical acceptance and regulatory approval for fetal or placental imaging applications.

### Advanced computational analysis in CEUS

#### Machine learning applications for perfusion quantification

Recent advances in artificial intelligence have revolutionized CEUS analysis capabilities, addressing the quantification challenges identified in earlier studies [55]. The integration of deep learning architectures with traditional perfusion modeling achieves diagnostic accuracies of 73 to 86% for placental pathology detection [56]. These advances represent a paradigm shift from subjective visual assessment to objective, reproducible quantification essential for clinical implementation.

The PlaNet-S model combines U-Net with SegNeXt transformer mechanisms for placental segmentation [57]. This architecture processes 512 × 512 pixel CEUS frames at 15 Hz through an encoder-decoder structure with skip connections and attention modules. Training on 2,060 annotated images from 103 patients achieves intersection over union scores of 0.73 compared to 0.68 for standard U-Net, with connected components accuracy improving from 56.7 to 86.0%. Inference time remains below 1 s per frame on NVIDIA RTX 3090 hardware, enabling real-time clinical application.

Long short-term memory networks process CEUS video sequences capturing wash-in and wash-out dynamics impossible with static imaging [58]. These networks analyze input sequences of 300 frames spanning 20 s at 15 Hz through three LSTM layers with 256 units each. Output includes time-intensity curve parameters and perfusion classification with 85.3% accuracy for distinguishing normal versus abnormal perfusion. The temporal analysis captures subtle perfusion variations missed by frame-by-frame assessment, particularly important for detecting early placental dysfunction.

Multi-model perfusion frameworks employ automated selection among five distinct models based on goodness-of-fit criteria [59]. The gamma-variate model utilizes the equation AUC = A·tᵅ·e^(-t/β) for standard perfusion curves. Lognormal distribution focuses on time-to-peak analysis, while the Local Density Random Walk model addresses heterogeneous flow patterns. First Passage Time analysis characterizes vascular architecture, and lagged normal distribution captures delayed enhancement patterns. Model selection requires Spearman correlation exceeding 0.95 with normalized root mean square error below 0.05, ensuring optimal fitting for diverse perfusion patterns.

#### In-silico modeling for protocol optimization

Computational models enable protocol refinement without patient exposure, addressing ethical constraints in pregnancy research [60]. Finite element models incorporate patient-specific placental geometry from MRI with 1.5 mm³ resolution. Tissue properties include Young’s modulus of 32 kPa for villous tissue and 2.29 MPa for decidua. Acoustic parameters assume sound speed of 1540 m/s with attenuation of 0.5 dB/cm/MHz. Microbubble dynamics follow modified Rayleigh-Plesset equations accounting for shell elasticity and gas diffusion. Validation demonstrates less than 10% deviation from experimental measurements, supporting model reliability for protocol optimization.

Monte Carlo simulations provide stochastic modeling of bubble behavior in maternal-fetal circulation [61]. These simulations track 10⁸ particles with measured size distributions through vascular networks modeled with fractal branching dimension of 2.7. Flow conditions incorporate maternal uterine flow of 600 mL/min and intervillous space flow of 140 mL/min. Output probability distributions for enhancement patterns enable dose optimization and timing protocol development. Applications include prediction of optimal injection-to-imaging delays and contrast dose requirements for specific clinical scenarios.

### Comparative analysis of ultrasound modalities in obstetrics

#### Technical capabilities and limitations

Comprehensive comparison reveals CEUS occupies a unique diagnostic niche balancing enhanced vascular visualization against increased complexity and cost [62]. Standard B-mode ultrasound achieves spatial resolution of 0.5 to 2.0 mm axially with minimal safety concerns, maintaining thermal index below 0.5 and mechanical index between 0.3 and 1.0. This modality serves as the foundation for routine obstetric imaging with costs of $50 to $100 per examination.

Color Doppler adds functional vascular assessment with spatial resolution of 1 to 3 mm and temporal resolution of 15 to 25 Hz. However, thermal indices increase to 1.0 to 3.0, requiring careful application particularly in first trimester scanning. Power Doppler provides angle-independent flow detection with enhanced sensitivity for slow flow, maintaining moderate safety profiles with mechanical index of 0.3 to 0.7. Three-dimensional and four-dimensional capabilities add volumetric assessment at 1 to 10 volumes per second, though often with inferior resolution compared to dedicated 2D imaging.

CEUS operates at uniquely low mechanical indices of 0.05 to 0.2 to prevent bubble destruction while achieving superior vascular detail through harmonic imaging and pulse inversion techniques. The technology detects vessels smaller than 100 μm diameter and provides real-time quantitative blood flow assessment rather than velocity-only measurements. The absence of angle dependence eliminates a major limitation of Doppler techniques. However, the $200 to $400 per study cost represents a 20 to 40 fold increase over standard examinations, with additional requirements for specialized training encompassing 8 to 16 h didactic instruction and 20 to 50 supervised examinations.

Equipment costs escalate significantly, with CEUS-capable systems costing $150,000 to $300,000 and software upgrades for existing platforms ranging from $10,000 to $25,000. Examination time extends to 30 to 45 min compared to 15 to 20 min for standard obstetric ultrasound. Personnel requirements include capability for intravenous access and monitoring, often necessitating additional staffing. The integration of multiple modalities in combined protocols, such as CEUS followed by Doppler reassessment or 3D volume acquisition with targeted 2D refinement, optimizes diagnostic yield but extends examination time to 45 to 60 min.

### Risk–benefit framework with quantitative analysis

#### Evidence-based decision algorithm

Quantitative risk assessment incorporating limited safety data with clinical severity reveals distinct indication categories [63]. High-priority indications with risk-benefit ratios below 0.1 include placenta accreta spectrum, where maternal mortality without diagnosis ranges from 3 to 7%. Standard ultrasound achieves sensitivity of 77 to 87%, with CEUS potentially adding 15% sensitivity improvement. The number needed to diagnose to prevent emergency hysterectomy is 3 to 5, while theoretical risk remains below 0.4% based on zero adverse events among 256 cases.

Severe fetal growth restriction with normal Doppler presents another compelling indication. Stillbirth risk reaches 15 to 20 per 1000 births, while Doppler sensitivity for placental insufficiency remains limited to 60 to 70%. CEUS adds value through detection of microvascular dysfunction invisible to Doppler, potentially optimizing delivery timing decisions. Twin-twin transfusion syndrome staging represents a moderate-priority indication with risk-benefit ratio of 0.1 to 0.5, given mortality exceeding 80% if untreated and potential for CEUS to improve staging accuracy.

Monte Carlo risk modeling using 10,000 simulations provides probabilistic assessment [64]. Input parameters include diagnostic accuracy with sensitivity of 85% and specificity of 90%, disease prevalence ranging from 1 to 5%, and adverse event probability following Beta distribution with α = 1 and β = 256. Output demonstrates positive expected utility for placenta accreta spectrum when prevalence exceeds 2%. Sensitivity analysis confirms robustness to ± 20% variation in input parameters, supporting clinical application in appropriately selected high-risk populations.

Low-priority or investigational indications with risk-benefit ratios exceeding 0.5 include routine screening in low-risk pregnancy, first-trimester applications during organogenesis, and direct fetal organ perfusion assessment. These applications require additional safety data before clinical consideration. The framework emphasizes multidisciplinary consultation for complex cases, comprehensive informed consent addressing experimental status and unknown long-term effects, and systematic outcome tracking through prospective registries.

### Future directions and a proposed roadmap for clinical translation

#### Immediate research priorities with specific endpoints

The path forward requires coordinated multicenter trials with clearly defined endpoints and standardized protocols. A Phase II safety trial planned for 2025 to 2027 should enroll 300 patients powered to detect 3% adverse event rate with 95% confidence interval. Primary endpoints include composite adverse events at 72 h, while secondary endpoints encompass comprehensive biomarker panels including troponin, creatinine, IL-6, and TNF-α. Stratification by trimester, indication, and contrast agent type will enable subgroup analysis essential for risk assessment.

Standardization initiatives through 2025 to 2026 should employ modified Delphi methodology [65]. Initial rounds will engage 50 international experts rating 45 protocol parameters, with subsequent refinement of parameters achieving greater than 70% agreement. Final consensus on core datasets should yield published guidelines for technique standardization, addressing the protocol heterogeneity currently limiting clinical translation.

Artificial intelligence validation studies from 2026 to 2028 require multicenter datasets of 5,000 CEUS examinations for external validation of automated analysis algorithms. Primary metrics include agreement with expert readers exceeding kappa of 0.8, with secondary outcomes assessing diagnostic accuracy for specific placental pathologies. These studies will establish whether AI-enhanced analysis can overcome current limitations in quantification and inter-observer variability.

A recent pilot study by Roberts et al. demonstrated promising preliminary results with 42 women undergoing CEUS between 24 and 36 weeks gestation [66]. Using standardized protocols with biomarker assessment including troponin and BNP, they detected 80% of placental lakes missed by B-mode imaging with no biomarker elevation and normal neurodevelopmental outcomes at 6 months follow-up. This represents the first study to include systematic biomarker monitoring and extended follow-up.

#### Critical knowledge gaps requiring investigation

Fundamental gaps prevent immediate clinical translation of CEUS technology for obstetric applications. Most critically, no formal meta-analyses exist specifically addressing obstetric CEUS, with the 256-patient evidence base scattered across heterogeneous case series lacking standardized protocols or systematic follow-up. First trimester safety data remains essentially absent, preventing assessment during the critical organogenesis period when teratogenic risks are highest.

Technical standardization represents an equally pressing challenge. Protocol variability encompasses contrast agent selection, dosing regimens ranging from 1.2 to 4.8 mL, imaging parameters spanning mechanical indices from 0.05 to 0.19, and analysis methods lacking standardized quantification software. The absence of pregnancy-specific analysis tools forces adaptation of software designed for solid organ assessment, potentially missing placental-specific perfusion patterns. Inter-observer variability of 10 to 20% even with experienced operators highlights the need for automated analysis and quality assurance metrics.

Long-term developmental outcomes remain entirely uncharacterized beyond the Roberts et al. pilot study [66], with most studies lacking follow-up beyond immediate neonatal periods. The theoretical risks of sub-clinical effects on neurodevelopment, particularly from first trimester exposure, demand comprehensive longitudinal assessment through early childhood. Priority research questions include establishment of dose-response relationships for contrast volume and mechanical index settings, comparative safety across different contrast formulations, and identification of vulnerable developmental windows.

Additional knowledge gaps include drug interactions with common obstetric medications, cumulative effects of repeat exposure in serial monitoring, and comparative effectiveness versus standard care through randomized controlled trials. The distinction between conventional microbubbles and emerging nanobubble formulations requires particular attention given different safety implications for placental crossing.

#### Professional society engagement and regulatory pathways

International societies including ISUOG, AIUM, and EFSUMB must develop consensus guidelines addressing patient selection criteria, imaging protocols, safety documentation requirements, and training standards [67]. These guidelines should build upon existing contrast ultrasound parameters while incorporating pregnancy-specific considerations. Regulatory pathways through FDA and equivalent international bodies require clear definition, potentially through compassionate use protocols initially before formal approval processes.

The establishment of prospective registries for systematic outcome tracking represents a critical infrastructure need. These registries should capture comprehensive maternal and fetal outcomes, extending through early childhood development. Standardized data collection protocols will enable pooled analyses essential for detecting rare adverse events and establishing safety profiles across diverse populations.

Recent initiatives include the HOPE Study protocol, a multicenter trial assessing uteroplacental vascularization in early first-trimester pregnancy using both CEUS and 3D power Doppler [68]. This represents the first prospective trial specifically designed to evaluate CEUS in first trimester, addressing a critical knowledge gap. Additionally, targeted CEUS applications for molecular imaging continue to evolve, with recent preclinical work demonstrating feasibility of detecting placental inflammation markers [69].

## Conclusion

Contrast-enhanced ultrasound represents a potentially transformative technology for maternal-fetal imaging, offering unique advantages in placental perfusion assessment while maintaining an encouraging preliminary safety profile. This comprehensive analysis reveals that while 256 pregnant women have undergone CEUS without clinically significant adverse events, fundamental methodological limitations preclude immediate clinical implementation. The heterogeneity in protocols, with mechanical indices ranging from 0.05 to 0.19 and frequencies from 2 to 9 MHz, combined with absent biomarker monitoring and long-term follow-up, demands systematic research investment before routine application.

The critical distinction between conventional microbubbles, which demonstrate safety through size-based placental exclusion with greater than 99% remaining in maternal circulation, and emerging nanobubble formulations with theoretical crossing potential via transcytosis pathways, represents a pivotal consideration for future development. This size-dependent safety profile, previously underappreciated in obstetric applications, must guide contrast agent selection and development. Recent advances in AI-enhanced analysis achieving 73 to 86% diagnostic accuracy through ensemble deep learning architectures offer solutions to quantification challenges that have historically limited CEUS adoption, yet require validation across diverse populations and imaging platforms.

The technology’s unique capabilities, including detection of vessels below 100 μm diameter, real-time perfusion quantification independent of angle, and purely intravascular distribution without tissue accumulation, position CEUS to address critical gaps in current obstetric imaging. For high-risk indications such as placenta accreta spectrum with 3 to 7% maternal mortality if undiagnosed, the calculated number needed to diagnose of 3 to 5 to prevent emergency hysterectomy presents compelling risk-benefit ratios supporting selective clinical application under appropriate ethical oversight.

The path forward necessitates coordinated multicenter trials enrolling minimum 1000 patients with standardized protocols, comprehensive safety monitoring including biomarker panels absent from current research, and long-term neurodevelopmental assessment extending through early childhood. Professional societies must develop consensus guidelines while regulatory bodies establish clear approval pathways. The integration of computational modeling for protocol optimization, machine learning for automated analysis, and systematic registries for outcome tracking will accelerate translation from investigational technique to evidence-based clinical tool.

Success requires not merely technical advancement but coordinated efforts spanning basic science, clinical research, regulatory development, and professional standardization. Only through such systematic efforts can CEUS transition from promising innovation to validated clinical practice, ultimately improving outcomes for high-risk pregnancies while maintaining the exemplary safety standards essential to obstetric imaging. The ultimate measure will be whether CEUS can deliver meaningful improvements in maternal and fetal outcomes that justify the increased complexity and cost compared to conventional ultrasound, a question that only rigorous prospective trials with appropriate endpoints can definitively answer.

## Data Availability

Not applicable. This is a review article and all data is sourced from previously published studies cited in the references.

## Abbreviations

AIUM: American Institute of Ultrasound in Medicine
ANGPT2: Angiopoietin-2
AT: Arrival time
AUC: Area under curve
BNP: Brain natriuretic peptide
CEUS: Contrast-enhanced ultrasound
CTG: Cardiotocography
CV: Coefficient of variation
EFSUMB: European Federation of Societies for Ultrasound in Medicine and Biology
FGR: Fetal growth restriction
FHR: Fetal heart rate
GBCAs: Gadolinium-based contrast agents
GD: Gestational day
HSP70: Heat shock protein 70
IL-6: Interleukin-6
ISUOG: International Society of Ultrasound in Obstetrics and Gynecology
IUGR: Intrauterine growth restriction
LSTM: Long short-term memory
MB-PS: Phosphatidylserine-targeted microbubbles
MCA: Middle cerebral artery
MI: Mechanical Index
MRI: Magnetic resonance imaging
MTT: Mean transit time
NICU: Neonatal intensive care unit
PAS: Placenta accreta spectrum
PI: Peak intensity
ROI: Region of interest
TI: Thermal Index
TIC: Time-intensity curve
TNF-α: Tumor necrosis factor-alpha
TTP: Time to peak
UAE: Uterine artery embolization
VEGF: Vascular endothelial growth factor
